# Diffusion tensor imaging in type 1 diabetes: decreased white matter integrity relates to cognitive functions

**DOI:** 10.1007/s00125-012-2488-2

**Published:** 2012-02-11

**Authors:** E. van Duinkerken, M. M. Schoonheim, R. G. IJzerman, M. Klein, C. M. Ryan, A. C. Moll, F. J. Snoek, F. Barkhof, M. Diamant, P. J. W. Pouwels

**Affiliations:** 1 Diabetes Centre/Department of Internal Medicine, VU University Medical Centre, de Boelelaan 1117, 1081 HV Amsterdam, the Netherlands; 2 Department of Medical Psychology, VU University Medical Centre, Amsterdam, the Netherlands; 3 Department of Radiology, VU University Medical Centre, Amsterdam, the Netherlands; 4 Department of Anatomy and Neuroscience, VU University Medical Centre, Amsterdam, the Netherlands; 5 Department of Psychiatry, University of Pittsburgh School of Medicine, Pittsburgh, PA USA; 6 Department of Ophthalmology, VU University Medical Centre, Amsterdam, the Netherlands; 7 Department of Physics and Medical Technology, VU University Medical Centre, Amsterdam, the Netherlands

**Keywords:** Brain, Cognition, Imaging, Microangiopathy, Type 1 diabetes, White matter tracts

*To the Editor*: Type 1 diabetes, particularly in the presence of microangiopathy, is associated with cognitive dysfunction, mainly observed in domains involving processing speed, suggesting white matter involvement [1]. White matter hyperintensities, a commonly used marker for white matter damage on MRI, however, do not occur more prevalently in type 1 diabetes compared with controls [2]. Therefore, we assessed white matter tract integrity using MRI-diffusion tensor imaging (DTI) and cognitive functions in type 1 diabetic patients with and without microangiopathy and in controls. We hypothesised that type 1 diabetic patients with microangiopathy would show the most pronounced reductions in white matter tract integrity compared with the other groups, and that these differences would be associated with cognitive differences.

Forty-eight patients with microangiopathy (mean age: 44.6 ± 7.3 years; mean HbA_1c_: 8.1 ± 1.3% [65.1 ± 14.4 mmol/mol]; mean disease onset age: 10.3 ± 7.1 years; mean disease duration: 34.3 ± 7.9 years; microalbuminuria: 29%; self-reported neuropathy: 52%), 52 patients without microangiopathy (age: 38.1 ± 9.1 years; HbA_1c_: 7.8 ± 0.9% [61.6 ± 9.9 mmol/mol]; disease onset age: 16.4 ± 9.6 years; disease duration: 21.7 ± 9.3 years) and 49 controls (age: 36.7 ± 11.2 years; HbA_1c_: 5.3 ± 0.2% [34.2 ± 2.6 mmol/mol]), matched for sex, IQ and BMI, were included. Inclusion and exclusion criteria, together with definitions of microangiopathy and severe hypoglycaemia, have been previously published [3]. Patients with microangiopathy were selected if they had proliferative retinopathy, but could also have other complications [3]. Those without microangiopathy had no clinically measurable complications. All participants filled out the Centre of Epidemiological Studies Scale of Depression [3], and routine blood and urine sampling was performed. Blood glucose during testing was kept between 4 and 15 mmol/l.

The neuropsychological assessment covered the following domains: general cognitive ability, memory, information processing speed, executive functions, attention, and motor and psychomotor speed [3]. MRI scanning was performed at 1.5 T (Siemens Sonata, Erlangen, Germany). DTI acquisition consisted of 10 volumes without directional weighting and 60 volumes with 60 non-collinear gradient directions (b-value 700 s/mm^2^), repetition time 8500 ms; echo time 86 ms; 59 contiguous axial slices, isotropic 2 mm resolution. DTI post-processing with FSL4.1 provided eigenvectors λ1, λ2 and λ3, and the derived parameters fractional anisotropy ([FA] general white matter integrity) and axial (diffusion parallel to the axon), radial (diffusion perpendicular to the axon) and mean (overall diffusion) diffusivity [4]. Tract-based spatial statistics (TBSS) were applied for FA [5], and for axial, radial and mean diffusivity. Voxel-based statistics with ‘randomise’ were corrected for multiple comparisons using the family-wise error (FWE) [6]. In case of an effect in all patients vs. controls, post hoc tests were performed with individual patient groups. Tractography was used to determine diffusion parameters in the bilateral corticospinal and inferior fronto-occipital tracts, as these showed the largest differences between patients and controls. Correlations between cognition and DTI parameters of these tracts were determined using linear regression. All analyses were corrected for age, sex, systolic blood pressure and depressive symptoms. A *p* value <0.05 (FWE-corrected) was considered to be statistically significant.

Patients with microangiopathy were significantly older and had more depressive symptoms and increased systolic blood pressure compared, albeit in the normal range, with the other groups (*p* < 0.05). Compared with those without microangiopathy, they had earlier onset age and longer disease duration (*p* < 0.05; electronic supplementary material [ESM] Table 1). TBSS analysis showed a widespread decreased FA in all patients vs. controls (Fig. 1a). Tracts most affected were the bilateral inferior fronto-occipital and corticospinal tracts, and the corpus callosum. Furthermore, decreased FA was observed bilaterally in the thalamic radiation, forceps minor and major, and superior longitudinal fasciculus. Patients with microangiopathy showed FA reductions in all these tracts, while FA reductions in patients without microangiopathy were limited to the corpus callosum and right corona radiata. Consequently, in many tracts FA was lower in patients with microangiopathy vs those without. In all patients, compared with controls, axial diffusivity showed a similar pattern of decrease to FA (Fig. 1b). Here, however, the most extensive decreases were found in patients without complications. Thus, the comparison between patient groups showed increased axial diffusivity in the corpus callosum in patients with microangiopathy vs. those without. Increased radial diffusivity was found in the posterior corpus callosum in all patients vs. controls (Fig. 1c). This was solely attributable to patients with microangiopathy, who showed increases in corpus callosum, bilateral corticospinal and inferior fronto-occipital tracts, forceps minor and major and corona radiata compared with both the other groups, whereas patients without microangiopathy did not differ from controls. Mean diffusivity did not show any alterations in patients vs controls. For all the above-mentioned comparisons, matching for age, diabetes duration, and onset age yielded similar results, despite inevitably reduced group sizes (ESM Fig. 1). In all patients, higher FA of the left corticospinal tract was related to better general cognitive ability and attention. Lower radial diffusivity of the left inferior fronto-occipital and corticospinal tracts were related to increased attention and executive functions and borderline with information processing speed. Lower axial diffusivity in the right inferior fronto-occipital tract was related to better psychomotor speed performance (ESM Fig. 2 a–f). In controls, no such correlations were found. These diffusion parameters were not related to life-time severe hypoglycaemic events.

**Fig. 1:**
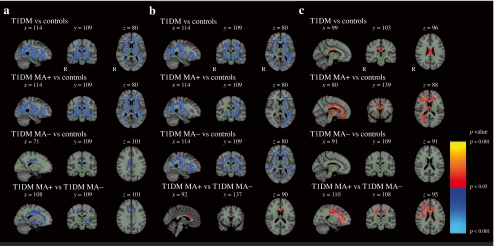
Visual representation of changes in FA (**a**), axial diffusivity (AD; **b**) and radial diffusivity (RD; **c**) for all groups. Red–yellow indicates an increase, whereas blue–light blue indicates a decrease (FWE-corrected *p* < 0.05). The mean skeleton is shown in green, and significant differences are displayed as thickened tracts for visualisation purposes. The *x*, *y*, *z* coordinates of the brains in Montreal Neurological Institute (MNI) standard space are given. T1DM MA+, type 1 diabetes patients with microangiopathy; T1DM MA−, type 1 diabetes patients without microangiopathy

The current FA results are comparable with one earlier small-sized DTI-study in type 1 diabetic patients [5], but, in addition, we showed that FA decrease is more extensive in patients with microangiopathy. We have extended these observations by the finding that spatially widespread reductions in axial diffusivity already occur in patients without microangiopathy and are most marked in those with microangiopathy, suggesting that axonal damage or loss of coherence in fibre bundles is an early process in type 1 diabetes [7]. The observed increase in radial diffusivity, occurring as microangiopathy develops, is thought to represent myelin damage [7]. As studies on the biological correlates of axial and radial diffusivity are performed in animals and not humans, the interpretation is still speculative. The current results do not support involvement of severe hypoglycaemia in these white matter tract changes. Simultaneous assessment of grey and white matter changes, as previously performed [8], would be an interesting future direction. Better cognitive performance was correlated with better white matter tract integrity in both patient groups. Longitudinal studies need to identify the course and (hyperglycaemia-related) underlying mechanisms of these diffusion changes as diabetes progresses.

## Electronic supplementary material

Below is the link to the electronic supplementary material.

ESM Fig. 1 PDF 101 kb

ESM Fig. 2 PDF 64 kb

ESM Table 1 PDF 11 kb

## Abbreviations

DTI: Diffusion tensor imaging
FA: Fractional anisotropy
FWE: Family-wise error
TBSS: Tract-based spatial statistics
