# Supplementary material for: Diffusion tensor imaging in type 1 diabetes: decreased white matter integrity relates to cognitive functions
Source: Diabetologia. 2012 Feb 11;55(4):1218–20. doi: 10.1007/s00125-012-2488-2 (PMC3296003; doi:10.1007/s00125-012-2488-2)
Supplement: Supplementary file 1 — PDF 101 kb [file 125_2012_2488_MOESM1_ESM.pdf]

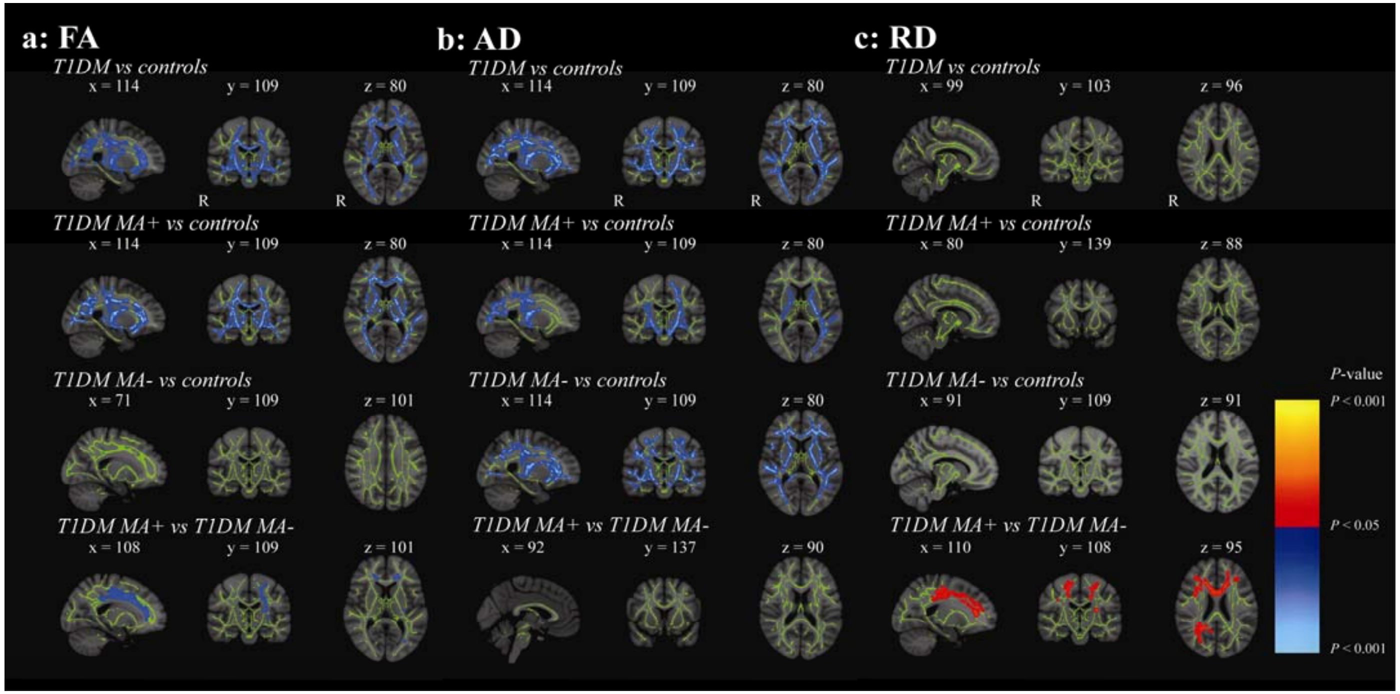

Figure 1. Visual representation of changes in fractional anisotropy (FA; a), axial diffusivity (AD; b), and radial diffusivity (RD; c) for all groups matched for diabetes duration and onset age, and current age. Red-yellow indicates an increase, whereas blue-light blue indicates a decrease (FWE-corrected $p<0.05$). The mean skeleton is shown in green, and significant differences are displayed as thickened tracts for visualization purposes. The x, y, z coordinates of the brains in Montreal Neurological Institute (MNI) standard space are given. T1DM MA+: type 1 diabetes patients with microangiopathy; T1DM MA-: type 1 diabetes patients without microangiopathy.

In this matched analysis, 27 of the 48 T1DM MA+ and 29 of the 52 T1DM MA- patients were included. All 49 controls were included. Despite this large reduction in sample size results for FA are similar, albeit the lower FA in patients without complications fails to reach significance with the conservative FWE-corrected test ($p=0.2$). Results for AD are also similar, although the increased AD in patients with complications relative to those without is not significant anymore. Results for RD are more influenced by the smaller sample size: there are no regions with statistically increased RD in all patients and patients with microangiopathy compared to controls. However, increased RD in patients with microangiopathy compared to their counterparts without microangiopathy remains statistically significant.
