# Supplementary material for: Diffusion tensor imaging in type 1 diabetes: decreased white matter integrity relates to cognitive functions
Source: Diabetologia. 2012 Feb 11;55(4):1218–20. doi: 10.1007/s00125-012-2488-2 (PMC3296003; doi:10.1007/s00125-012-2488-2)
Supplement: Supplementary file 2 — PDF 64 kb [file 125_2012_2488_MOESM2_ESM.pdf]

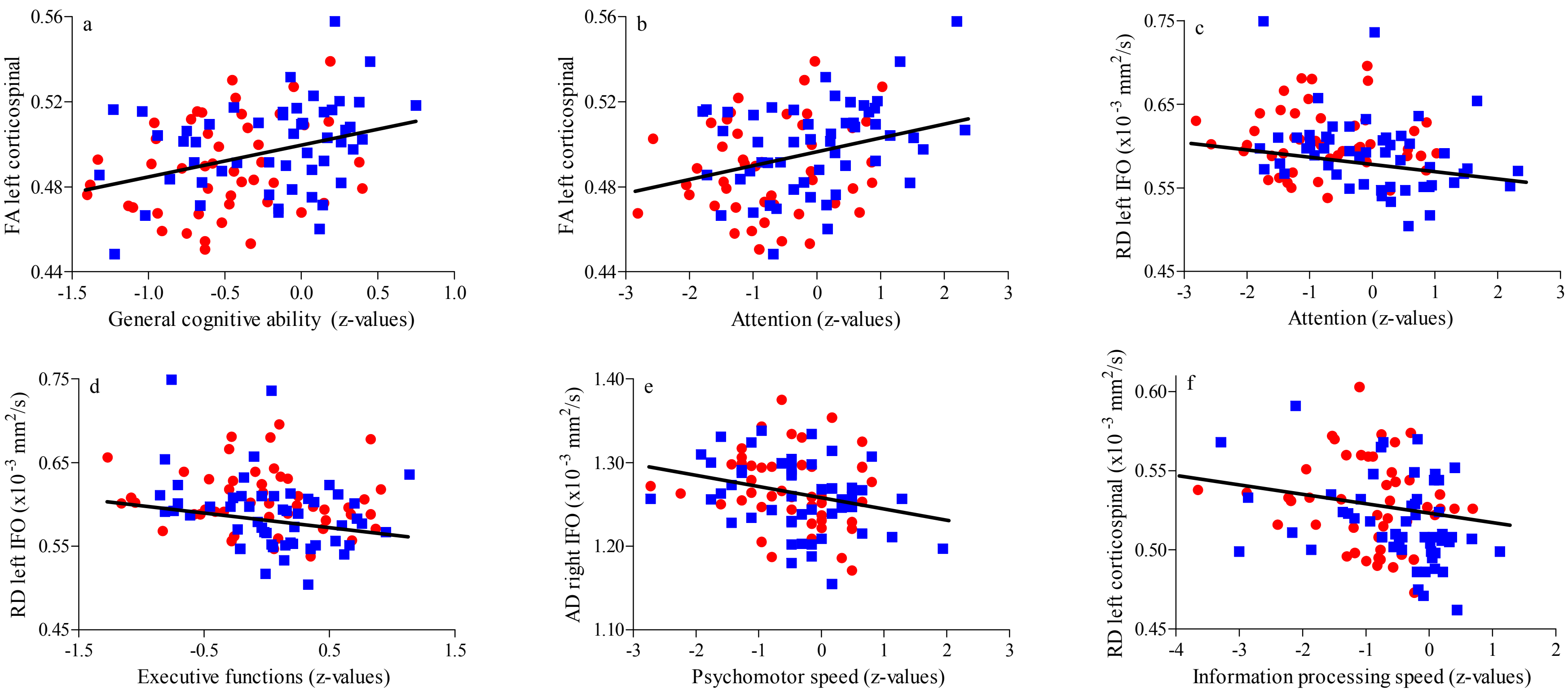

Figure 2a-f. Scatter plots of associations between DTI parameters (fractional anisotropy FA, radial diffusivity RD, and axial diffusivity AD) and cognitive domains. red circles represent patients with microangiopathy and blue squares denote patients without complications. Correlations were only calculated for the T1DM patients and the standardised regression coefficient is given for the whole group. Correlations are corrected for age, sex, systolic blood pressure, depressive symptoms and diabetes duration.

a: β = 0.230, p = 0.016; b: β = 0.259, p = 0.008; c: β = -0.219, p = 0.031; d: β = -0.208, p = 0.049; e: β = -0.220, p = 0.036;
f: β = -0.184; p = 0.076.
