# Supplementary material for: Diffusion tensor imaging in type 1 diabetes: decreased white matter integrity relates to cognitive functions
Source: Diabetologia. 2012 Feb 11;55(4):1218–20. doi: 10.1007/s00125-012-2488-2 (PMC3296003; doi:10.1007/s00125-012-2488-2)
Supplement: Supplementary file 3 — PDF 11 kb [file 125_2012_2488_MOESM3_ESM.pdf]

Table 1. Demographic, anthropometric and medical variables of T1DM patients with microangiopathy, T1DM patients without microangiopathy, controls.

	T1DM with microangiopathy	T1DM without microangiopathy	Controls	*p* value
N	48	52	49	-
Age (years)	44.6 ± 7.3	38.1 ± 9.1	36.7 ± 11.2	<0.001
Sex (m/f)	19/29	20/32	20/29	0.997
Body Mass Index (kg/m^2)	25.6 ± 4.2	24.9 ± 3.5	24.3 ± 3.6	0.224
Estimated IQ[a]	110.5 ± 13.5	106.8 ± 11.2	108.5 ± 11.7	0.310
Depressive symptoms[b]	11.1 ± 9.7	7.0 ± 6.6	6.3 ± 6.7	0.005
Systolic blood pressure (mmHg)	134.0 ± 17.0	129.3 ± 15.0	123.6 ± 11.3	0.003
Diastolic blood pressure (mmHg)	75.9 ± 8.5	77.9 ± 9.4	77.4 ± 7.2	0.189
HbA1c (mmol/mol)	65.1 ± 14.4	61.6 ± 9.9	34.2 ± 2.6	<0.001
HbA1c (%)	8.1 ± 1.3	7.8 ± 0.9	5.3 ± 0.2	<0.001
Updated HbA1c[c] (mmol/mol)	65.5 ± 12.4	61.3 ± 8.3	-	0.067
Updated HbA1c[c] (%)	8.1 ± 1.1	7.8 ± 0.8	-	0.067
Hypertension[d]	31 (64.6)	13 (25.0)	-	<0.001
Albumin-to-creatinine ratio (mg/mmol)	3.9 ± 6.5	0.6 ± 0.6	-	<0.001
Blood glucose before MRI (mmol/l)	9.2 ± 3.8	10.3 ± 4.6	-	0.189
Blood glucose before NPA[e] (mmol/l)	8.7 ± 4.1	8.4 ± 4.1	-	0.718
Diabetes duration (years)	34.3 ± 7.9	21.7 ± 9.3	-	<0.001
Diabetes onset age (years)	10.3 ± 7.1	16.4 ± 9.6	-	0.001
Severe hypoglycaemic events[f]	6.1 ± 9.3	5.8 ± 10.1	-	0.872
Neuropathy[g] (%)	25 (52.1)	-	-	-
Microalbuminuria[h] (%)	14 (29.2)	-	-	-

Values are given as mean with standard deviation or absolute numbers with percentages between parentheses.

[a] Estimated IQ was measured using the Dutch version of the National Adult Reading Test (NART).

[b] Depressive symptoms were self-reported using the Centre for Epidemiological Studies scale for Depression (CES-D).

[c] Updated HbA1c from the period of 2002 until study date. Median period was 7 years.

[d] Hypertension was defined as a systolic blood pressure of 140 mmHg or above, a diastolic blood pressure of or above 90 mmHg, or the use of antihypertensive medication.

[e] NPA is neuropsychological assessment.

[f] Severe hypoglycaemic events are those events for which a patient needs a third person to recuperate from low blood glucose levels, seizure or coma. These events were self-reported.

[g] Neuropathy was self-reported or, in case of doubt, ascertained by the patients medical record.

[h] Microalbuminuria was defined as an albumin-to-creatinine ratio >2.5 for men and >3.5 for women.
